# How policy promotes the integration of culture and tourism? A fuzzy-set qualitative comparative analysis based on the Policy Instrument Theory

**DOI:** 10.1371/journal.pone.0301119

**Published:** 2024-04-29

**Authors:** Yunyun Tang, Kaigang Yi, Zijian He

**Affiliations:** 1 School of Tourism and Urban-Rural Planning, Zhejiang Gongshang University, Hangzhou, China; 2 School of Business Administration, Zhejiang Gongshang University, Hangzhou, China; Hungarian University of Agriculture and Life Sciences: Magyar Agrar- es Elettudomanyi Egyetem, HUNGARY

## Abstract

Crafting pertinent policies to facilitate the high-level integration of culture and tourism has now become a vital agenda within the current discourse in China. However, relatively little is known about the actual implementation of various policies to achieve a high-level integration, especially how combinations of policy instruments are deployed in the process of realization. Based on the Policy Instrument Theory, this study uses fuzzy-set qualitative comparative analysis on a sample of 31 provincial administrative regions in China to investigate the influence of typical policy instruments on the integration level of tourism and culture. The results show that each single policy tool is not necessary for high-level integration of culture and tourism. On the contrary, only through an organic combination of different policy tools can affect the integration level. This study also summarizes five policy instrument configurations, which can be grouped into four driving modes of culture-tourism integration: the environment-driven supply-demand coordination mode, supply-driven demand-environment coordination mode, supply-driven mode, and supply-driven environment coordination mode. This study considerably provides critical theoretical and practical insights into the integration of culture and tourism from the perspective of governmental policies.

## Introduction

Integration of culture and tourism is China’s most effective path for adapting to the new requirements of cultural industry and tourism development in the new era [1, 2]. Starting from 2009, the Chinese government has been actively implementing a range of policies to stimulate the integration of culture and tourism. The “Guidance on Promoting the Combination of Culture and Tourism” released in August of the same year by the former Ministry of Culture and the former National Tourism Administration can be viewed as the first official policy for this purpose. Over the course of the subsequent years, more than 80% of provincial governments have introduced their own localized integration policies. The formal establishment of the Ministry of Culture and Tourism in 2018 marked the recognition of culture-tourism integration as a strategic initiative driven by the national level. It is widely acknowledged that policy levers play a crucial role in guiding and propelling its construction and development [3, 4].

When it comes to policies, we can find extensive previous studies have closely linked public or special policies to the development of cultural and tourism industries at various levels, such as Pearce [5], Zhang, Chong and Ap [6], Lai, Li and Feng [7], and Haigh [8]. A common conclusion of these studies lies in that relevant policies formulated by the government have been affirmed as an essential tool to effectively promote the development of cultural and tourism industries. However, some studies hold a conservative view that policies may not always produce positive effects, as various subjective and objective factors can discount the effectiveness of policy implementation [9, 10]. With respect to both positive and discounted impact of policies on cultural and tourism industries, we find that the current econometric methods dominate these studies. Among them, the computable general equilibrium model [11, 12] and difference-in-differences method are commonly used in policy analysis [3, 13]. By scrutinizing panel data or time-series data, scholars have effectively showcased how policies profoundly affect industrial progress. Concurrently, they have substantially enhanced the understanding of the intricate relationship between policies and industries. However, in spite of these great efforts, obvious shortcomings also exist.

Current econometric analyses identify the single conditional variable of policy’s role in increasing the outcome variable. For instance, Matteo [14] evaluated the “National Strategy for the Inner Area” program’s effectiveness in Italy, and found that this policy significantly impacted extra-hotel accommodations. Zhang, Singh and Yu [15] also demonstrated that the vacation policy approved in 2007 changed the demand for tourism in mainland China. Additionally, governmental policy is typically treated as one of the independent variables influencing the development of cultural and tourism industries, which is subjected to quantitative testing alongside other factors. Based on the benevolent DEA cross-efficiency model, Lu, Ren and Zhai [16] explored six critical factors’ influence on the integration efficiency of culture and tourism, and made a surprising conclusion that policy environment played a significant negative impact. Yang, Zhou and Li, et al. [17] indicated that there was a configurational effect of policies and other factors on the convergency of sports industry and tourism industry. In a word, these studies have identified the varying impact of policies. However, we note that existing literature usually view the policy as a whole and only focus on its overall effect. The “black box” of policy mechanism remains unexplored, meaning that we still do not fully understand why policy itself can exert effects. Policy is a set of action plans or guidelines formulated by authoritative institutions. It normally includes main goals, basic principles, explicit tasks, working methods, general steps, and specific measures within a certain historical period. Deconstructing policy to explore the role of key contents or points seems more meaningful. An important consideration is to examine the relationship of various policy points from a configurational perspective.

Based on these findings, we propose the research gaps are as follows: First, scholars acknowledge the critical role of policy in the development of cultural industry, tourism industry, and their integration. Many studies, however, naturally treat policy as a whole rather than considering its internal structure and focal points. Second, researchers exhibit an interest for assessing the actual effectiveness of policies embraced by local governments. However, they have allocated notably insufficient attention to the specific policy points, as well as their resultant effectiveness. Integration of culture and tourism is an inevitable trend in the global industrial development. In the context of China, the government plays a leading role in promoting the integration of these two industries. It leads us to ask: what integration policy instruments are selected and adopted by each province in China? what is the effectiveness of different combinations of policy points in terms of high-level integration?

To answer these questions, this study adopts the Policy Instrument Theory to break down existing integration policies. This theory enables the categorization of policy texts into various policy instruments [18, 19]. We extract three categories of policy instruments, namely the supply-oriented, demand-oriented, and environmental-oriented ones by combing the existing literature. Subsequently, we assess the integration level of culture and tourism in 31 provincial administrative regions in China through a constructed evaluation index system. To further explore how the combination of different policy instruments affect integration performance, we conduct a fuzzy-set qualitative comparative analysis (fsQCA) based on a configurational perspective and discuss the fsQCA results. The fsQCA believes that it is the combination of conditions rather than individual conditions alone that leads to the emergence of the outcome. Its aim is to uncover the intricate interactions among conditions when the outcome occurs [20–22]. This method explores the “multiple conjunctural causation” relationships formed by different combinations of antecedent conditions [23], which can help identify the diverse policy-driven integration pathways of culture and tourism. Therefore, the main characteristics of the fsQCA method make itself very applicable in this study, thus persuading us to employ it to do our research.

The main contribution of this study is fourfold. In theoretical construction, we employ a comprehensive analytical framework for integration policies and accordingly interpret them to multiple policy instruments. From this standpoint, we posit that both the presence and absence of policy instruments, along with their combinations, serve as the driving forces behind integration quality. This compensates for the insufficiencies in prior policy research. Methodologically, in contrast to conventional econometric approaches, we employ an fsQCA method to holistically explore and reveal the necessary and sufficient conditions for integration performance. Regarding the empirical cases, we select 31 provinces, municipalities and autonomous regions in China, primarily based on the several considerations. On one hand, while the level of integration varies across provinces, they share a common primary goal. This alignment adheres to the fsQCA criteria of achieving both “maximum similarity” and “maximum heterogeneity” among cases [23]; on the other hand, as mentioned earlier, due to the diverse realities in different regions, the policy effectiveness varies during their formulation and implementation process. Hence, taking the 31 provincial-level administrative regions as research cases is conducive to a comprehensive understanding of the impact of Chinses integration policies on integration performance. Besides, the identified policy-driven pathways in this study can also provide reference and guidance for the practice of culture-tourism integration.

We organize this study into six sections. Section 2 presents the review of key literature and theoretical analytical framework. Section 3 follows with research methods, data collection and processing strategies. Section 4 and 5 respectively show the fsQCA results and discussion. The last section concludes with the main contributions, limitations, and future research.

## Literature review

### Integration of culture and tourism

Although the integration of culture and tourism has not yet formed a unified concept, “cultural tourism” has become an internationally recognized alternative expression corresponding to it [24, 25]. Since the 1980s, several western countries have been paying attention to the relationship between cultural heritage and tourism, as well as the relevant concepts. In 1985, the United Nations World Tourism Organization (UNWTO) made a broad and narrow distinction in defining “cultural tourism”. With the continuous evolution of the relationship between culture and tourism, relevant organizations such as the UNWTO and the European Association for Tourism and Leisure Education (ATLAS) have been actively promoting cultural tourism, emphasizing and harnessing the attractive power of culture in tourism. In academia, cultural tourism has been interpreted from a deeper perspective. For instance, Richards [25] believed that cultural tourism constitutes a type of tourist activity wherein the fundamental motivation of travelers is to learn, explore, experience, and consume both tangible and intangible cultural attractions or offerings in the destination. These attractions or products encompass a range of material, intellectual, spiritual, and emotional characteristics of a society, including art and architecture, history and cultural heritage, cuisine, literature, music, creative industries, and more. Building upon this foundation, cultural tourism has given rise to related concepts such as heritage tourism [26], and cultural creative tourism [27]. Unlike Chinese concept of “融合” (integration), the UNWTO uses the term “synergy” to emphasize the importance of interactive integration between cultural industries and tourism institutions. This was explicitly described in the document “tourism and culture synergies” in 2018 [16, 28].

Typically, people often perceive the cultural industry based on a fixed understanding of culture. In a narrower sense, cultural industry encompasses various cultural content sectors such as music, dance, literature, painting, film, animation, gaming, etc., covering diverse domains within the realms of literature and art [29, 30]. In contemporary society, the rise of creative economy has broadened the scope of cultural industry beyond its traditional confines. It now includes numerous emerging fields closely related to cultural creativity [31], reaching into industries such as manufacturing, agriculture, commerce, tourism, and even everyday life. The “interdisciplinary” nature of the cultural industry allows it to transcend the boundaries of multiple sectors and engage in cross-cutting interactions with various related industries [30]. This characteristic creates exciting possibilities for the integration of culture and tourism. Although cultural industry and tourism industry are two different sectors, the fundamental reason why they can be integrated is possible lies in their respective industrial characteristics. Specifically, tourism has dual economic and cultural attributes, and its cultural benefits can be realized through the development of industrial economy, while culture can also achieve commercialization through reasonable development and utilization [32, 33]. The cross-border integration of culture and tourism can be seen as a dynamic and win-win relationship. They engage in a two-way interaction, mutually influencing and benefiting from each other. As these two industries converge, they intertwine and recombine their elements, gradually surpassing the constraints of their original boundaries. Consequently, industry borders shrink, blur, or even disappear, giving rise to innovative cultural and tourism product formats and a new, more flexible industry system.

Research on the integration of culture and tourism primarily involves several aspects, such as the modes, impact, evaluation, and influencing factors. Cultural heritage tourism serves as a typical example, and it has been extensively studied. Among these studies, the conservation of cultural heritage resources and their development for tourism purposes have remained a popular topic of discussion in academia [34, 35]. For numerous cultural heritage sites along the Silk Road in China, cultural tourism has been proven to be a viable measure for their management [36]. In recent years, cultural creativity has played a driving role in the transformation and upgrading of tourism industry, accelerating the integration of culture and tourism [27]. This has led to the emergence of numerous cultural tourism industrial parks [37] and cultural tourism performance products [38]. In order to achieve the organic combination of modern consumption and distinctive culture, cultural tourism towns, exemplified by Wuzhen Town, are springing up in China [39]. As for coastal regions such as Shanghai and Fujian, the development of marine cultural tourism has more advantages [40, 41].

Under this backdrop, the reciprocal influence between cultural and tourism industries exhibits both positive and negative dimensions [42]. On one hand, the integration fosters increased technological demands within industries, leading to industrial innovation [43]. Additionally, it also contributes to the preservation and dissemination of traditional culture [44] while significantly increasing tourism performance [3]. On the other hand, it may pose a threat to the authenticity of culture [45]. The commercialization process could introduce detrimental impacts on cultural aspects [46]. According to Zeng, Shen and Gu’s latest research findings [47], there is a single-threshold effect, indicating that a positive impact is only evident when the integration of culture and tourism reaches an elevated level. Currently, the conceptual scope and industrial boundaries of culture and tourism continue to expand, leading to new requirements for measuring and evaluating the integration level. Scholars often assess the performance of culture-tourism convergency from two key indicators: coupling coordination [48, 49] and efficiency [50, 51]. The variations in the integration outcomes highlight the practical significance of studying its influencing factors, which include the level of economic development, traffic conditions [16], human capital [52], resource endowment [53], financial environment [54], and technological changes [55]. Furthermore, it also depends on the role of governments and their policy interventions [56]. As such, clarify the existing policy research becomes necessary to gain insight into the actual issue of culture-tourism integration in a real-world context. It helps to point out the direction of optimization and adjustment in policy design for industrial development.

### Policy of culture-tourism integration

As various forms of cultural tourism continue to flourish, countries like United States, Japan, and South Korea have proactively introduced relevant policies to develop cultural tourism [25]. Against this backdrop, foreign scholars have shown early interest in the policy issues concerning their own country’s cultural and tourism industries, which include topics related to employment [57], poverty reduction, and cultural promotion [58]. In a highly centralized regime like China, the development of local cultural tourism relies on policies formulated by the central government for support. Several scholars have comprehensively analyzed the progression of Chinese cultural and tourism-related policies, employing policy literature quantitative analysis as a methodology, such as Tang [58], and Gao, Ryan and Zhang, et al. [4].

In addition to specific policies, the effectiveness of policy implementation is another crucial issue. Logar [59] explored the potential efficacy of eight sustainable tourism policy instruments and proposed three criteria for policy evaluation, namely effectiveness, acceptability, and economic-technical feasibility. Kim and Kim [60] indicated that cultural tourism represents a crucial policy measure for regional development, thereby contributing to the broader goal of revitalizing regional economy. However, policies do not always yield positive outcomes. Kouri [61] examined the integration practice of culture and tourism in Greece from a government policy perspective. They discovered that these policies resulted in increasing tensions between socio-cultural and financial objectives, which has drawn the attention of international policy makers. Recently, researchers have paid increasing attention to the empirical evaluation of policies on the integration of culture and tourism [8]. A study from China demonstrates that not all cities are suitable for implementing the policy of culture-tourism integration, as the policy performance may be poor in regions where the cultural industry is significantly less developed than the tourism industry [47].

The heterogeneity of policy effectiveness makes the discussion of influencing factors more sense. The government is the primary policy maker, and political factors significantly influence policy formulation and implementation [9]. The effectiveness of policies relies on the support of economic forces. Hence, when formulating policies, it’s essential to take into account the economic advantages and national economic contribution. Deller [62] found that in more developed regions, policy implementation is more effective. Additionally, regional resources, such as cultural norms [63], natural resources [64], human capital [65], and technological infrastructure [10], have been demonstrated to play a critical role in the implementation of cultural and tourism industry policies.

Given the complexity of policy documents, many scholars employ the Policy Instrument Theory to extract key points and summarize policy texts. From the perspective of public policy studies, policy instruments refer to the means, methods, and measures chosen by government and other decision-making entities to achieve predetermined objectives [18]. Its essence lies in the transformation of overarching public management goals into concrete actionable rules. There is a certain degree of variation in the categorization of policy instruments among researchers. Ma, Jong and Bruijne, et al. [66] made a classification of low carbon instruments into hierarchy-based, market-based, network-based and information-based instruments. The policy instruments of Perman, Ma and McGilvray, et al. [67] could be divided into three types, namely economic, regulatory and institutional instruments. Following on this research, Logar [59] had explored the potential use of eight such instruments for managing more sustainable tourism. As research advances, the content of the Policy Instrument Theory has gradually become more comprehensive, encompassing a diverse range of tools, criteria for categorization, classification frameworks, and value objectives [68, 69]. Existing research largely refers to and draws upon the fundamental typology of policy instruments defined by Rothwell and Zegveld [70], which comprises three categories: supply-side, demand-side and environmental instruments. Scholars have further interpreted and subdivided the general typology mentioned above, proposing numerous distinct subcategories based on their research themes and content [66, 70].

From previous research, it is evident that the Policy Instrument Theory is frequently applied to decode policy texts and conduct statistical comparisons of the quantities of different policy instruments. The identification and selection of core policy instruments plays a guiding role in promoting the high-quality integration of culture and tourism. This, in turn, facilitates policymakers in gaining a deeper understanding of policy processes, the interplay and impacts of policy instruments, thereby providing a robust basis for subsequent policy designs. This notion has been tested by numerous tourism scholars [59, 71] and can serve as the analytical framework for this study.

### The analytical framework based on the Policy Instrument Theory

**Fig 1:**
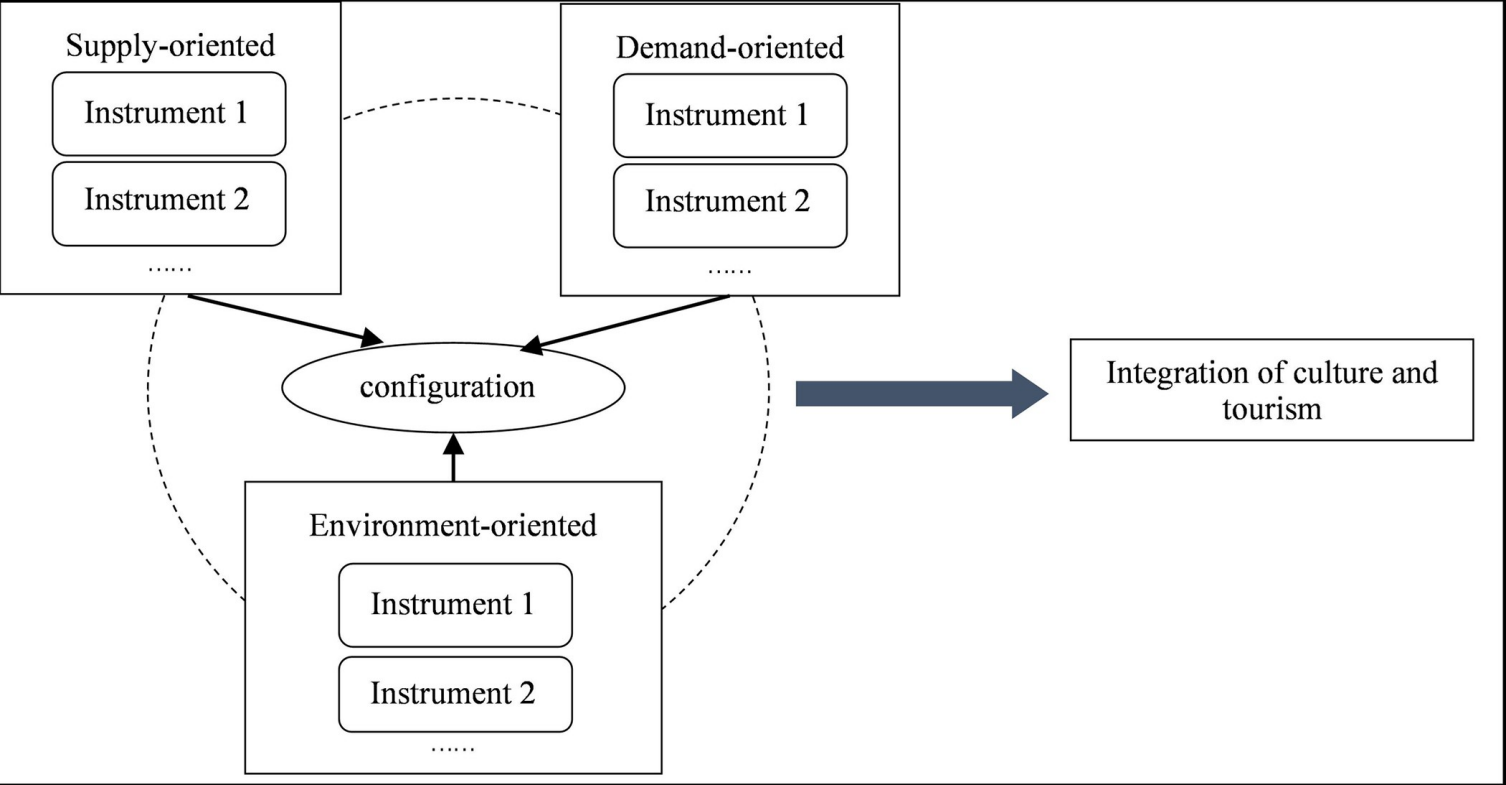
The conceptual model of policy instruments influencing the integration of culture and tourism.

Based on Rothwell and Zegveld’s research [70] and taking into account the actual context of culture-tourism integration in China, policy instruments in this study mainly be categorized into three types: supply-oriented, demand-oriented, and environment-oriented instruments. In accordance with previous research findings and the specific content of policy documents, a more detailed sub-categorization is performed to construct a comprehensive analytical framework for our study. The above mentioned three types of policy instruments complement each other and collectively contribute to the integration of cultural and tourism industries (Fig 1).

Specifically, supply-side policy instruments refer to the government’s strategic focus on providing fundamental infrastructure and public services necessary for the integration. These instruments involve targeted investments in critical production factors such as talent, funding, technology, and land, offering various foundational guarantees and support to increase the integration level. Examples of supply-oriented policy instruments include infrastructure construction, public cultural services, financial incentives, technological advancements, and talent development initiatives.

Demand-side policy instruments entail the government’s direct interventions to stimulate demand within the cultural and tourism industries to facilitate their development. These instruments mainly refer to a range of stimulating policies, including government-led initiatives for specialized cultivation and pilot projects. Moreover, they involve initiatives to promote domestic and international exchanges and cooperation, facilitating active participation from various societal stakeholders. Additional measures consist of strategies to encourage overseas trade, boost consumer spending, and implement comprehensive promotional campaigns. By collectively augmenting demand, these policy instruments aim to foster the growth and advancement of the cultural and tourism sectors.

Environment-oriented policy instruments pertain to the governmental endeavors aimed at establishing a conducive policy landscape for the harmonized growth of cultural industry and tourism sectors. These instruments are designed to facilitate policy implementation and enforcement, expedite market acceptance and response to policies, and indirectly impact the growth of cultural and tourism enterprises. They encompass the formulation of industry development goals and plannings, establishment of diverse regulations and administrative supervision measures, as well as initiatives concerning COVID-19 pandemic prevention and control, among others.

## Methodology

### Data collection

#### Policy texts selection

The official establishment of the Ministry of Culture and Tourism in 2018 marked a significant milestone in elevating the integration of culture and tourism to the national strategic level. The increasing attention to it is evident in the growing number and intensity of related policy documents. Therefore, this paper defines the window period for integration policies of culture and tourism as spanning from 2018 to 2021.

To gather relevant data, we employed keywords such as “cultural tourism(文化旅游)” and “integration of culture and tourism(文旅融合)” to conduct comprehensive searches across various relevant government official websites. Several selection criteria are followed to ensure the representativeness and accuracy of the policy texts: (1) The policy documents included originate from provincial-level government institutions and their respective functional departments. Specifically, we only consider policy documents released by various Provincial People’s Government Offices, Department of Culture and Tourism. To maintain the focus on provincial-level policies, we exclude texts that are forwarded from national-level authorities by provincial administrative regions. Additionally, policy documents from municipal levels under each province’s jurisdiction are not considered for this study. (2) The policy texts are strictly relevant to the theme of integration. Policy documents that merely mention “strengthening the integration of culture and tourism” or similar generalized statements without substantial content are excluded. Besides, we carefully eliminate policy texts with titles or themes related to culture or tourism but lacking concrete content and measures regarding their integration. (3) The policy texts mainly consist of laws, regulations, ordinances, detailed implementation rules, methods, plans, opinions, notices, working points, and other similar types of documents. Those with lower relevance, such as industry standards, official replies, letters, training materials, conference notifications, policy interpretations, announcements, press releases, and other related documents, are excluded from the analysis. (4) Any ones that have already expired or unavailable for download are also not considered. After thorough screening, a total of 149 policy documents were selected for inclusion in this study’s analysis.

#### Index system of the integration of culture and tourism

In this study, the integration level of culture and tourism is selected as the outcome variable indicating the effectiveness of policies. The evaluation index system for the integration level comprises 15 secondary indicators, categorized into four dimensions: industrial efficiency, industrial structure, industrial performance, and industrial influence (as shown in Table 1). Due to the time lag in the impact of policies, this study used the evaluation scores for the 31 provincial regions in China in the year 2021 as the raw data. To highlight the integration aspect, this study gives equal importance to both culture and tourism when selecting indicators, aiming for a roughly equal number of indicators related to each. The data for the indicators were sourced from the “China Cultural Heritage and Tourism Statistics Yearbook 2022”, “China Cultural and Related Industries Statistics Yearbook 2022”, as well as the statistical yearbooks of various provinces and cities for the year 2021. Additionally, some data were obtained from the National Economic and Social Development Statistical Bulletin. It should be noted that the regional cultural and tourism attention index comes from the annual average search index of the Baidu Index platform. As for the index “average expenditure per overnight inbound tourist” for the year 2020, the average value of data from 2017, 2018, and 2019 is used as a substitute. For individual missing data, linear interpolation was used for supplementation.

**Table 1:** Evaluation index system for the integration level of culture and tourism.

Dimensions	Indicators (Measurement Units)	Interpretation
Industrial efficiency	Revenue per employee in the culture and related industries (10,000 yuan per person)	Labor productivity of employees in the cultural and tourism industry
	Total tourism revenue per employee in the tourism industry (10,000 yuan per person)	
	The ratio of income from cultural and related industries to fixed asset investment in the tertiary sector (%)	ROI of the cultural tourism industry
	The ratio of total tourism revenue to fixed asset investment in the tertiary sector (%)	
Industrial structure	Employment ratio in culture and related industries to tertiary sector (%)	Employment scale of cultural tourism industry
	Employment ratio in tourism industry to tertiary sector (%)	
	Cultural and related industries revenue as a proportion of regional GDP (%)	The economic status of the cultural tourism industry in the region
	Total tourism revenue as a proportion of regional GDP (%)	
Industrial performance	Number of visitors to major cultural institutions (in hundred thousand person-times)	Reception scale of cultural tourism industry
	Total tourist arrivals (in hundred thousand person-times)	
	Revenue of culture and related industries (in hundred million yuan)	Revenue scale of cultural tourism industry
	Total tourism revenue (in hundred million yuan)	
	Operating profit of cultural and related enterprises above designated size (in hundred million yuan)	Profitability of Cultural Industry Enterprises
	Average expenditure per overnight inbound tourist (in US dollars per person-day)	Foreign exchange earning capacity of tourism enterprises
Industrial influence	Regional cultural and tourism Baidu search index	The dissemination and influence of the cultural tourism industry

In economics, industrial efficiency mainly pertains to the ratio of industrial input to output, which measures the capability and extent of transforming a certain input element into output [72]. Broadly speaking, workforce and fixed assets are regarded as fundamental elements of production within the realms of both tourism and cultural industries [73]. Tourism stands as a labor-intensive sector, with its economic operations significantly influenced by the workforce it employs [74]. According to Chinese Tourism Statistical Yearbook, A-grade scenic spots, star-rated hotels, and travel agencies are the most important components within tourism industry, with their employees constituting the primary labor resource. Therefore, the combined number of employees from the above sub-sectors as the labor input metric for the tourism industry. While the most straightforward indicator for gauging labor elements in cultural industry would be the count of people employed, the relevant statistical yearbook lacks comprehensive data. Thus, the number of employees in cultural and related corporate entities is selected as an alternative substitute. Fixed asset investment and economic growth maintain a durable and steady symbiotic relationship of mutual promotion over the long term [75]. As we all known, both cultural industries and tourism industries belong to the tertiary sector. In this study, fixed asset investment in the tertiary sector is used as the capital input index. As for the measurement of output, economic benefits serve as the optimal indicator. We apply the total revenue from tourism and cultural industry to represent [76]. Combining input factors with output results, revenue per employee in the cultural and tourism industries, and the ratio of total industrial revenue to fixed asset investment in the tertiary sector are respectively selected as the representation for industrial efficiency.

In this study, the structural aspects of cultural and tourism industries are mainly reflected in the employment structure and income structure. The proportion of employment in the cultural and tourism sectors concerning the overall national economy serves as an indicator of their employment-generating potential. Considering the unique attributes of these industries, and drawing upon the work of Gao, Yan and Du [77], we have chosen to represent the employment structure of cultural and tourism industries by calculating the ratio of their respective number of employees to the total number of employees in the tertiary sector. Based on the findings of Zhang’s study [31], the proportion of industry revenue to GDP is an important indicator of the position and role of an industry in the national economy. Accordingly, this research has selected two indicators, namely the percentage of cultural and related industry revenue, as well as total tourism revenue to regional GDP.

Total industry revenue mirrors the level of industrial development and its industrial status, while total recipients showcase the impact and outreach stemming from industry progress [78]. The combination of these two metrics stands as the prevailing indicator for evaluating the performance of industrial production endeavors [79]. Therefore, we select tourism revenue and revenue of culture and related industries as well as tourist arrivals and the number of visitors to major cultural institutions as the main indicators of industrial performance. Furthermore, operating profit of cultural and related enterprises above designated size has been chosen to evaluate the profitability of cultural enterprises [80]. In essence, tourism industry is an export-oriented sector and a significant source of foreign exchange income for our country. The average expenditure of overnight inbound tourists is positively correlated with the economic strength and tourism development of various regions. In this paper, it is considered one of the indicators for measuring industry performance [81]. Ultimately, in order to evaluate industrial outward influence, the Baidu search index of regional cultural and tourism is used [82].

### Research method

**Content analysis.** Content analysis is a quantitative research method used for systematic and objective analysis of textual data to identify and measure specific themes, patterns, and trends within the text [83]. This method enables textual data to be encoded into quantifiable values through a word-by-word and sentence-by-sentence analysis [84]. This study is based on a database comprising 149 policy texts related to the integration of culture and tourism. Utilizing the Nvivo 12 software and referencing the previous policy instrument analysis framework, the policy texts were meticulously deconstructed, read, analyzed, annotated, and coded on a sentence-by-sentence basis. Each policy item was categorized in line with its content and primary keywords, and the occurrence frequency of each policy sub-tool was subsequently summarized according to the respective provincial regions. To ensure the credibility of the coding results, the entire coding process was independently conducted by two researchers. In cases of coding discrepancies, a third expert was consulted to provide judgment. As a result, a total of 22 policy tool reference points were obtained (Table 2), with a coding reliability of 92.363%.

**Table 2:** Examples of policy text coding and overview of policy tools.

Subcategories		Description	Frequency
Supply-oriented instruments			
1	Infrastructure construction	Improving water, electricity, roads, transportation, information networks, and other necessary infrastructure construction for cultural and tourism activities.	1082
2	Public services	Promoting the integration of culture and tourism from the perspective of public services, such as public library services and museum visits.	882
3	Financial input	Providing financial support to the supply side through special funds, grants, and fiscal measures to promote the development of cultural and tourism.	464
4	Technical support	Providing information technology and data support for cultural and tourism integration to promote the reform of the supply side.	1165
5	Talent support	Providing talent support for cultural and tourism integration, such as talent training and talent introduction.	902
6	Land support	Supporting cultural and tourism integration through measures such as land use indicators allocation, encouraging the revitalization of land resources, and optimizing land approval procedures.	303
Demand-oriented instruments			
7	Government procurement	Government purchasing of cultural and tourism products and services, stimulating the demand for the development of related industries	65
8	Special cultivation or pilot project	Developing specialized cultivation plans and programs for the cultural and tourism industry, and piloting the construction of exemplary projects.	519
9	Exchange and cooperation	Initiating cultural and tourism exchange and cooperation projects with other regions, institutions, and organizations both domestically and internationally.	1021
10	Overseas trade	Encouraging inbound tourism and overseas exports of cultural and tourism products to create international demand for the cultural and tourism industry.	38
11	Boosting consumption	Stimulating cultural and tourism consumption through the cultivation of related product formats, free access to related venues, and distribution of consumption vouchers.	1053
12	Promotion and publicity	Strengthening promotion and advertising, establishing a brand system, organizing marketing events, and other methods to increase visibility.	1727
13	Platform construction	Building relevant development platforms, such as digital information platforms, exchange and cooperation platforms, etc.	131
Environment-oriented instruments			
14	Goals and planning	The macro-level development planning and goal-setting for the process of cultural and tourism integration.	3296
15	Epidemic prevention and control	Measures taken to prevent and control the COVID-19 pandemic, such as crowd management and disinfection.	54
16	Supervision and regulation	Regulation, supervision, and enforcement for the cultural and tourism market and activities, including standardization and penalties.	1874
17	Financial policy	Providing policy support for financing, investment attraction, and subsidized loans in the cultural and tourism market.	320
18	Incentives and subsidies	Incentives through bonuses, subsidies for cultural and tourism projects, and tax benefits for cultural and tourism enterprises.	335
19	Intellectual property	Intellectual property rights protection for cultural and tourism products.	49
20	Industrial synergy	Promoting the coordinated development between the cultural and tourism industry and other related industries.	572
21	Organizational support	Strengthening leadership mechanisms, enhancing organizational support and administrative supervision, and promoting interdepartmental collaboration.	661
22	Institutional reform	Administrative management system reform and adjustments undertaken to promote cultural and tourism integration.	235

Generally, the higher the occurrence frequency of a policy tool, the greater the attention it receives from relevant government departments, indirectly indicating its significance. Following the requirements of the fsQCA method for the number of conditional variables, for a more effective research outcome in small sample-sized studies (10 to 40 cases), it is advisable to limit the model to a maximum of 7 condition variables [23]. Therefore, in this study, policy sub-tools with a frequency of less than 1000 were excluded, and a final selection of 7 specific policy instruments, namely “infrastructure construction”, “technical support”, “exchange and cooperation”, “boosting consumption”, “promotion and publicity”, “goals and planning”, and “supervision and regulation” were chosen as conditional variables for fsQCA analysis.

### Multivariate linear composite index method

This study employed the multivariate linear composite index method to evaluate the integration level of culture and tourism. One of the key steps is to determine the weights of each indicator. Commonly used methods for weight determination include subjective and objective weighting techniques [85, 86]. Subjective weighting methods mainly rely on researcher’s personal experience and judgment, with weight assignment exhibiting strong subjectivity. In contrast, objective methods use the original data itself for calculation, examine the internal differences of data samples, and do not consider the theoretical importance of the indicators. Consequently, the weight assignment is more objective and scientific. Based on the entropy method in this study, the weights of each indicator were obtained [86], and the comprehensive scores of the integration level were calculated for each provincial-level administrative region. The measurement model is as follows:

$$F_{i}=a_{1}X_{i1}^{\prime }+a_{2}X_{i2}^{\prime }+\ldots \ldots +a_{j}X_{ij}^{\prime }$$ (1)

Where: *F_i_* represents the comprehensive score of the *i*-th province, $X_{ij}^{\prime }$ denotes the standardized value of the the *i*-th province under the *j*-th indicator, *a_j_* signifies the weight of the *j*-th indicator.

### fsQCA

fsQCA is a configurational approach based on set-theoretical perspective [20, 23] and Boolean algebra algorithms [87]. It is very suitable for studying complex causal relationship and the resultant configurational effects [88]. The method claims the outcome variable (the integration level of culture and tourism in this study) is the result of the combined effect of multiple conditional variables (different policy instruments in our case). Two main indicators (consistency and coverage) were used to assess the fit between the conceptual model and the empirical data [89]. Consistency evaluates the degree to which the observed evidence supports the presumed relationships within the model. Meanwhile, coverage describes the explanatory power of conditions (or combinations of conditions) on the outcome variable. Both indicators range from 0 to 1, with higher values reflecting greater reliability in the calculated results. Their formula is as follows:

$$Consistency(X_{i}\leq Y_{i})=\frac{\sum min(X_{i},Y_{i})}{\sum X_{i}}$$ (2)

$$Coverage(X_{i}\leq Y_{i})=\frac{\sum min(X_{i},Y_{i})}{\sum Y_{i}}$$ (3)

Where: *X_i_* and *Y_i_* represent the membership degree of unit *i* in the condition set *X* and outcome set *Y*, respectively.

**Fig 2:**
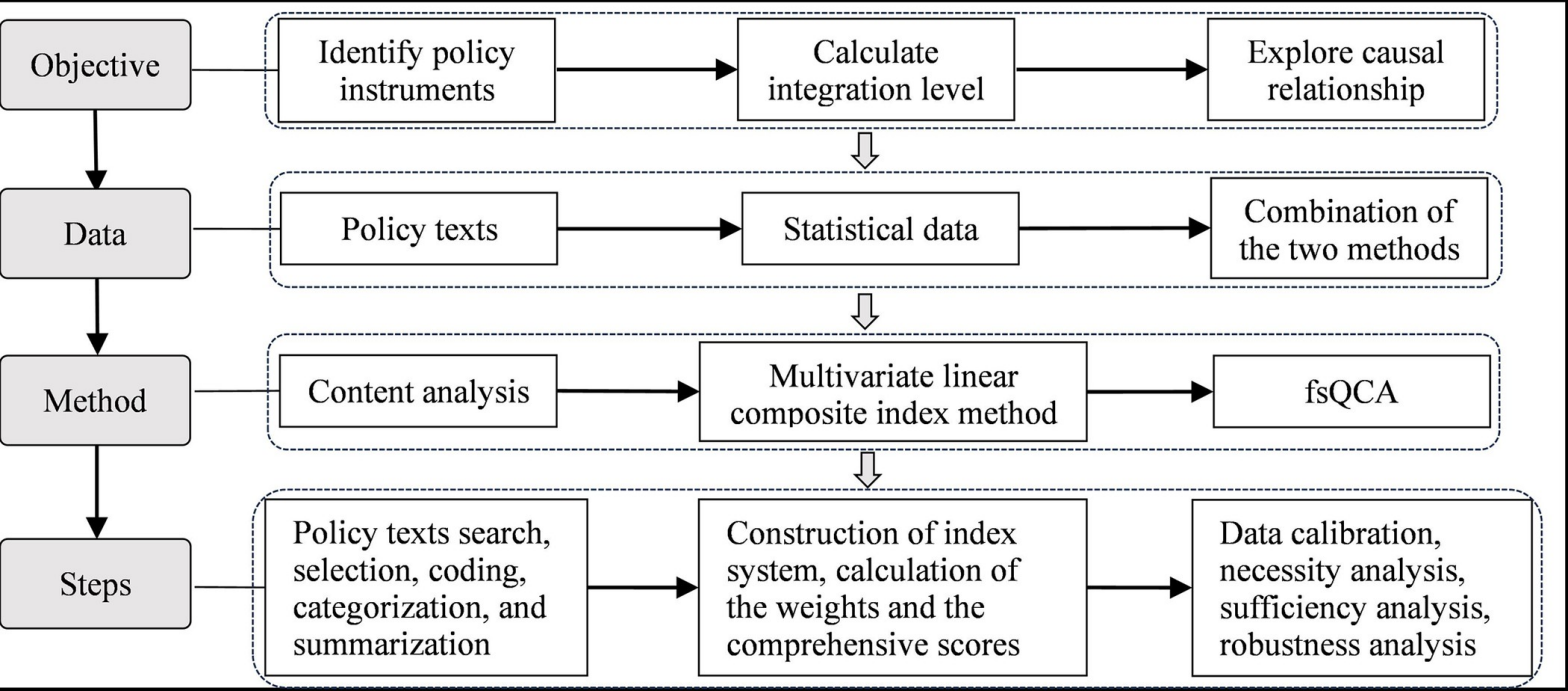
Research process of this study.

Data in this study were analyzed by the fsQCA3.0 software. The entire analysis comprises four steps, with detailed instructions for each step provided in the following sections. Fig 2 illustrates the research process in this study.

## Findings

### Results

#### Data calibration

In fsQCA, the initial step involves the calibration of the original variable values by transforming them into sets and assigning distinct membership degrees between 0 and 1. Two common methods for data calibration are direct calibration and indirect calibration, both of which require the identification of three anchor points: the full membership, crossover point, and full non-membership. Drawing from existing research [90, 91] and considering the data attributes and characteristics, this study adopted the direct calibration method. Specifically, the full membership, crossover point, and full non-membership for the seven conditional variables and one outcome variable were set as the 90th percentile, 50th percentile, and 10th percentile of the sample data, respectively. The descriptive statistics and calibration anchor points for each variable are summarized in Table 3. For the convenience of writing, we use the abbreviations of eight variables to represent (infrastructure construction (IC); technical support (TS); exchange and cooperation (EC); boosting consumption (BC); promotion and publicity (PP); goals and planning (GP); supervision and regulation (SR); culture-tourism integration (CTI)).

**Table 3:** Descriptive statistics for each variable and calibration anchor points.

Variable	Descriptive statistics				Calibration anchor points		
	Mean	SD	Min	Max	Full membership	Crossover point	Full non-membership
Infrastructure construction (IC)	34.903	16.841	2	78	55	36	16
Technical support (TS)	37.581	23.149	1	100	70	33	12
Exchange and cooperation (EC)	32.935	19.205	3	79	59	28	11
Boosting consumption (BC)	33.968	24.436	0	85	64	32	5
Promotion and publicity (PP)	55.710	30.967	12	130	97	49	20
Goals and planning (GP)	106.323	55.986	1	231	191	98	49
Supervision and regulation (SR)	60.452	38.208	5	135	122	52	13
Culture-tourism integration (CTI)	0.266	0.149	0.068	0.627	0.508	0.286	0.095

### Necessary conditions analysis

Prior to analyzing the configurational effects of conditional variables on outcome variable, it is crucial to examine the necessity of individual conditions (including both present and absent states) [92, 93]. This entails determining whether a specific condition is a necessary requirement for the occurrence of outcome. In terms of set relationships, this is manifested when the outcome set is a subset of a particular condition set. Generally, a condition is considered “necessary” when its consistency value exceeds 0.9 [94], which should be retained in the subsequent sufficiency analysis of condition configurations. Table 4 shows the necessity analysis results. From this, each consistency value is less than 0.9, denoting that all antecedent conditions are not necessary for high-level integration of culture and tourism.

**Table 4:** Necessity analysis of a single condition.

Conditional variable	Outcome variable	
	Consistency	Coverage
IC	0.537	0.497
~IC	0.673	0.531
TS	0.676	0.591
~TS	0.616	0.511
EC	0.648	0.540
~EC	0.583	0.508
BC	0.639	0.568
~BC	0.578	0.472
PP	0.609	0.519
~PP	0.665	0.567
GP	0.523	0.477
~GP	0.680	0.543
SR	0.629	0.545
~SR	0.637	0.533

Note: “~” represents the logical operation “not”, meaning “does not exist”.

### Sufficiency analysis of condition configurations

This analysis aims to reveal different combinations of conditions that produce specific results, which in set theory is equivalent to determining whether the set of condition configurations is a subset of the set of outcomes. The fsQCA3.0 software was used to construct a truth table based on the calibrated fuzzy membership scores. In order to retain valuable and stable condition combinations and exclude “noisy” ones, a frequency threshold of 1 [93] and a consistency cut-off of 0.8 were set [23]. At the same time, it was ensured that the Proportional Reduction in Inconsistency (PRI) of the configuration was greater than 0.65 [95, 96]. By refining and solving the truth table, three types of solutions were finally obtained: the complex solution, intermediate solution, and parsimonious solution. The complex solution is faithful to the original case data and does not include any logical remainders, usually containing the most configurations and antecedent conditions. The parsimonious solution includes all logical remainders but does not evaluate their rationality, and has the fewest configurations and conditions. The intermediate solution only includes logical remainders that are consistent with theoretical explanations and practical experience, with moderate complexity. It is usually the preferred reporting and interpretation approach in QCA research [97]. Therefore, we choose the intermediate solution as the basis for interpreting the results, while combining the parsimonious solution to identify the core and peripheral conditions of the given configuration. Conditions that appear in both parsimonious and intermediate solutions are identified as core ones, indicating a strong causal relationship with the results. On the contrary, peripheral conditions only appear in intermediate solution with a weaker role in the outcome compared to core conditions.

As shown in Table 5, there are five configurational paths influencing the integration of culture and tourism. The overall solution with a consistency of 0.773 and a coverage of 0.501, implying that this solution accounts for 50.1% of the membership in the outcome. These five paths are sufficient conditions lead to desirable outcome in this study.

**Table 5:** Configurations driving the integration of tourism and culture.

Conditional variable	Condition configurations				
	H1	H2	H3	H4	H5
IC	●	●	●	●	⊙
TS	●		●	●	●
EC	⊙	●	⊙	⊙	⊙
BC	●	●	●	⊙	⊙
PP		●	●	⊙	⊙
GP	⊙	⊙		⊙	●
SR	●	●	●	⊙	●
Consistency	0.852	0.811	0.792	0.808	0.927
Raw coverage	0.231	0.292	0.253	0.176	0.192
Unique coverage	0.012	0.088	0.025	0.034	0.046
Solution consistency	0.773				
Solution coverage	0.501				

Note: For solid black circle (●) denotes the presence of a condition; concentric hollow circle (⊙) indicates its absence. Large circle represents the core condition, small circle suggests the peripheral condition. A blank space means that the condition is unimportant for that configuration.

### Robustness analysis

Robustness analysis is also known as sensitivity analysis, which is used to examine whether research results are robust under the conditions of using other discriminant criteria. The commonly used methods include three types: changing calibration anchors, adjusting consistency thresholds, and adding or removing cases [93]. In this study, we adopted the second method and adjusted the PRI consistency threshold of 0.65 to 0.7 [98, 99]. The obtained configuration paths are consistent with the original ones, and the overall consistency and coverage of the solutions remain the same. Based on the fitted parameters and set relationship status, it can be considered that the results of this study are robust.

### Interpretation

Overall, the five paths can be summarized into four modes, namely the environment-driven supply-demand coordination mode, supply-driven demand-environment coordination mode, supply-driven mode, and supply-driven environment coordination mode. The specific details of each mode are as follows:

(1) Environment-driven supply-demand coordination mode. The core conditions of this mode belong to environment-oriented policy instruments, while the peripheral conditions consist of supply-side and demand-side policy tools, corresponding to the configuration paths H1 and H2.

The path H1 can be described as IC*TS*~EC*BC*~GP*SR, indicating that regardless of the measures of publicity and promotion, as long as the environment-oriented policy instruments led by governmental supervision and regulation can work together with favorable supply-oriented policy tools, high integration performance can be produced, even if exchange and cooperation, as well as goals and planning is lacking. Based on the pathway analysis above, it can be deduced that at the provincial level in China, integration policies emphasize the government’s service-oriented role and focus on supply-side optimization and reforms. This configuration has the highest consistency at 0.852 and account for 23.1% of the membership. The solution IC*EC*BC*PP*~GP*SR is defined as H2, which suggests that governmental planning is rare and technological input doesn’t contribute to integration performance. However, desirable results can be achieved with the combination of high-level of regulation, favorable demand-side policies, and certain infrastructure support. It is the work of many provinces who dominate this coverage of 29.2%, implying that such mode is relatively universal in China. Both configurations are centered around environment-oriented policy instruments, with H1 being mainly supported by supply-side policy tools, while the impact of demand-side policy tools is relatively weaker. On the other hand, in H2, all demand-side policy tools are present and exert positive influence, whereas the importance of supply-side policy tools is comparatively lower.

The typical cases that fulfill the configuration of H1 and H2 are Zhejiang Province and Yunnan Province, respectively. Zhejiang Province has introduced a series of benchmarking measures for the development of cultural and tourism industries. These measures cover the construction and regulation of various projects, such as tourism-themed towns, leisure tourism demonstration villages, and cultural and tourism intellectual property (IP) initiatives. Meanwhile, Yunnan Province has strategically positioned itself as a center radiating towards South Asia and Southeast Asia. It has been actively promoting regional cooperation, promotion, exhibition, and brand image to support its role in the “Belt and Road” construction.

(2) Supply-driven demand-environment coordination mode. The core conditions are supply-side policy tools, with peripheral conditions belonging to demand-side and environment-oriented ones. This configuration corresponds to H3, which can be denoted as IC*TS*~EC*BC*PP*SR. In this mode, government places high importance on technological investments in the development of cultural and tourism industries. Cultural and tourism industries remain deeply integrated through policy measures such as infrastructure construction, consumption stimulation, publicity and promotion, and supervision and regulation, even though without external collaboration and cooperation. This solution has the least consistency of 0.792 with a coverage at 25.3%. We can observe that all supply-oriented policy instruments play a positive role, while demand-side and environment-oriented tools have relatively weaker driving forces, represented by Hubei Province. The Hubei provincial government has taken various initiatives in response to the impact of the COVID-19 pandemic. By introducing preferential policies that benefit the public and providing assistance to cultural and tourism enterprises, Hubei Province has been continuously releasing the potential of cultural and tourism consumption, contributing to the recovery and growth of tourist market.

(3) Supply-driven mode. As the name suggests, supply-oriented policy tools are the core condition of this path, corresponding to the configuration H4. This path is depicted as IC*TS*~EC*~BC*~PP*~GP*~SR. It indicates that despite lacking policy support for collaboration, consumption stimulation, publicity, specific planning, and supervision, effective integration of culture and tourism can be obtained through substantial investment in technology and infrastructure. This solution covers 17.6% of the cases. In this configuration, only supply-side policy tools play a role, while the driving forces of demand-side and environment-oriented policy tools are not significant. Chongqing Municipality can be classified to this mode. Due to its distinctive geographical location and terrain, Chongqing Municipality encounters challenges in infrastructure construction. In recent years, the municipal government has been committed to promoting pilot projects for the development of new urban infrastructure. Focusing on information technology, digitization, and intelligent solutions, Chongqing is at the forefront of elevating urban development through smart city infrastructure upgrades.

(4) Supply-driven environment coordination mode. In this mode, the supply-oriented policy tools are core conditions, with supporting conditions being environment-oriented ones. We define this configuration as ~IC*TS*~EC*~BC*~PP*GP*SR, showing that high-level integration of culture and tourism will occur through increasing investment in information technology, implementing effective goals and planning, and strengthening supervision and regulation, even if there are deficiencies in other policy measures. This solution has the highest consistency at 0.927 and account for 19.2% of the membership. The typical case is Sichuan Province. As a leading demonstration area for digital cultural tourism, Sichuan Province is committed to innovative development of new cultural and tourism formats through digital technology. It has successively introduced a series of policy documents, outlining clear development goals and guiding principles for culture-tourism integration, and has established specific safeguards and management systems.

## Discussion

The five configurations identified in this study, although each composed of different condition variables, all propose a high-level integration of culture and tourism. Actually, they can be classified into two types based on the same set of core conditions (SR*~GP and TS*~EC, respectively).

The first one with high supervision and regulation, and few goals and plannings is consisted of two sub-configurations, namely H1 and H2. Considering China’s unique administrative regime, top-down environment-oriented policy instruments are widely adopted by local governments. Such regulatory instruments can yield relatively straightforward effect and are comparatively simple to deploy. In line with recent studies that have expressed support toward the role of governmental oversight and control in boosting industrial development [66], the findings confirm that the tool of supervision and regulation plays a core role in the integration of culture and tourism. However, in itself, the tool of supervision and regulation is sufficient. We find that the absence of goals and plannings is the premise for the effectiveness of it. That is, we should weaken the role of planning tools in the process of industrial integration, which correlates with previous views that government’s role in industrial planning has undergone a transition from control to consultation [100, 101]. Meanwhile, mandatory tools such as supervision and regulation can have an immediate and significant impact, considerably counteracting the gradual effects of planning and goals, thus to some extent realize the replacement of them.

The second type includes three sub-configurations (H3, H4 and H5, respectively), which means the combination with high-intensity of technical support and less exchange and collaboration can lead to a deep industrial integration. The role of information technology in industrial productivity [55], alongside with government’s preferences [102], making it a primary supply-side tool in government policies. Contrary to previous findings in their emphasis and attention to policies of exchange and cooperation in industrial integration [103], this study suggests that the policy tool of exchange and cooperation is likely to impose a negative influence on the integration of culture and tourism. In a word, it is unlikely for technology- and cooperation-based policy instruments to coexist in a single policy configuration. Technology-related policy tools directly improve the utilization and allocation efficiency of production elements [104], which in turn, reduces the motivation for regional exchange and cooperation. Conversely, frequent and in-depth communication and collaboration can achieve the flow of elements between countries and regions, facilitate the sharing of technology and knowledge [105, 106]. This may weaken the importance of government’s technological investment to a certain extent.

Upon comparing the five configurational paths, it is evident that H2 exhibits the highest coverage. This implies that a majority of 31 provincial regions achieve high-level integration of culture and tourism through H2, reaffirming the pivotal role of multi-dimensional coordination among various types of policies. In terms of co-existing policy tools, infrastructure construction suggests a high contribution in most configurations, which aligns with the findings of Tóth, Dávid, and Vasa [107] who found a multiple and complex relationship between transport and industrial development. It is noteworthy that the sub-tools “goals and planning” and “exchange and cooperation” have shown limited impact across all five paths, appearing only once. This might be attributed to their lack of immediate effectiveness. Despite being frequently mentioned in government policies, their actual influence on promoting the integration of culture and tourism remains relatively weak.

## Conclusions

In China, the integration of cultural and tourism industries is highly valued at both the national and provincial levels. In this context, this study applied fsQCA to a sample of 31 provincial administrative regions in China and examined the impact of combinations of integration policy instruments on the level of culture-tourism integration.

The findings indicate that China’s provincial culture-tourism integration policy tools can be categorized into three major types: the supply-oriented, demand-oriented, and environmental-oriented, totaling 22 sub-tools. Among them, there are 7 typical policy tools with occurrence frequencies exceeding 1000. An analysis of the policy instrument configurations shows their application cannot independently constitute the necessary conditions for high-level integration. Only through an integrated perspective and organic combination of different policy tools can they maximize their effectiveness. The fsQCA results demonstrate that there are four policy-driven paths for the integration of culture and tourism, which encompass five different configurations. The instrument tool combination of high supervision and regulation, and few goals and plannings (SR*~GP), as well as high-intensity of technical support and less exchange and collaboration (TS*~EC) are the key conditions to promote industrial integration.

Conclusively, the theoretical contribution of this study is considerable. This paper employs configurational thinking to analyze the synergistic effects among different policy instruments. It offers a systematic perspective and theoretical exploration, facilitating understanding of the policy-driven mechanisms behind the integration of culture and tourism in China, which has not been realized by previous studies. Besides, it refines and expands the Policy Instrument Theory and Qualitative Comparative Analysis (QCA) techniques in the fields of tourism studies and public policy analysis, thereby enriching the relevant theoretical research on the topic of culture-tourism integration. Furthermore, it provides essential theoretical support for policy transformation and optimization in the context of deep integration of cultural and tourism industries. It serves as a valuable reference for promoting further integration and mechanism innovation in China’s cultural and tourism practices, aligning with the strategic background of the new development stage of “Chinese-style modernization”.

The main practical implications are as follows: Firstly, this study advocates for optimization of the combination and distribution of policy instruments. It suggests increasing the formulation of demand-side and supply-side policies so as to reasonably allocate the proportion of policy instruments. What’s more, findings highlight that policies related to culture-tourism integration have been predominantly centered around planning and blueprints, lacking more actionable measures. Hence, it recommends optimizing the use of specific policy sub-instruments of different types in the current policy documents. Secondly, developing an effective policy evaluation system is crucial. Through a comprehensive review of existing policies at the provincial level, it becomes evident that many policies share high levels of duplication and lack uniqueness. Moreover, there is a significant absence of content related to policy evaluation. As a result, the cultural and tourism departments need to establish a robust policy execution and feedback mechanism, clearly delineating the roles and responsibilities of all relevant stakeholders. Additionally, proactively involving third-party evaluation agencies and leveraging the advisory role of think tanks can provide valuable, specialized insights and recommendations for optimizing policy formulation. Last but not least, special consideration should be given to demand-driven policy instruments. In this study, the overall utilization of demand-oriented policy tools is relatively limited, and they all exist as non-core conditions. Among the five configurations, only “boosting consumption” appears three times. Conversely, various supply-oriented policy tools are not only abundantly referenced in policy texts but also frequently appear in the configurational paths. Given this observation, the government needs a shift in mindset from relying solely on supply-oriented policies to proactively guiding and addressing the demands of market, which can also stimulate and foster the integrated development of cultural and tourism industries.

We conclude our study by addressing some improvements. First of all, it made use of second-hand policies and documents originated from official websites of various provincial government departments in China. Despite efforts to ensure the coding validity of policy texts, the presence of certain degree of subjectivity and biases is inevitable. Furthermore, data availability constraints have impacted the completeness of the constructed index system for assessing the integration level of culture and tourism, which necessitates further refinement in future studies. Additionally, the fsQCA method used in this research places specific requirements on the number of antecedent conditions. In this study, the selection of only seven policy sub-tools highlights the need for future investigations to explore the effects of other policy tools. Moreover, an essential direction for future research lies in quantitatively assessing the effectiveness of policy implementation.

## Supporting information

S1 Data (XLSX)

S2 Data (XLSX)
